# CCorGsDB: a database for clock correlated genes in the mouse and human central nervous systems

**DOI:** 10.1038/s44323-025-00064-y

**Published:** 2026-01-09

**Authors:** José Luiz Araújo Santos, Vinícius Tenório Braga Cavalcante Pinto, Thales Eduardo da Silva Santos, Daniel Gomes Coimbra, Tiago Gomes de Andrade

**Affiliations:** Circadian Medicine Center, Faculty of Medicine, Federal University of Alagoas, Maceió, Alagoas Brazil

**Keywords:** Biomarkers, Computational biology and bioinformatics, Neuroscience

## Abstract

We developed CCorGsDB, a web-based resource integrating mouse and human CNS co-expression networks filtered by circadian biomarkers. We validated the networks using time-series data from sixteen mouse regions and identified a conserved set of 251 orthologs enriched for RNA processing, chromatin regulation, and autism-related phenotypes. The database links CCorGs to disease terms and short–half-life CNS-active drugs with spatially resolved profiles. CCorGsDB is available at https://famed.ufal.br/ccorgs.

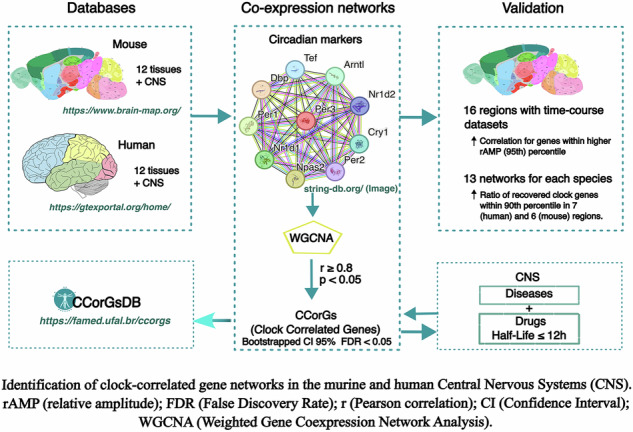

## Introduction

Circadian rhythms modulate essential physiological and behavioral processes in mammals and are increasingly recognized as key determinants of brain health and disease^1–4^. While the molecular clock machinery and its core genes are well characterized, how these oscillations shape gene regulation across specific brain regions remains only partially understood.

Although the suprachiasmatic nucleus (SCN) functions as the central pacemaker^5^, numerous extra-SCN brain regions exhibit autonomous rhythmic activity^6^. These local oscillations are thought to underlie the temporal coordination of neural and behavioral functions, and their disruption is implicated in diverse neuropsychiatric conditions^7,8^. However, the molecular components driving these region-specific rhythms in the central nervous system (CNS) remain poorly characterized.

Because clock genes are broadly expressed^9^, but clock-controlled genes display high tissue and cell-type specificity^10^, identifying circadian-regulated transcripts in distinct CNS regions is challenging. The scarcity of time-series transcriptomic data in human brain tissue further constrains this effort^3,11–19^. Additionally, rhythmic function does not always coincide with rhythmic transcription—post-transcriptional mechanisms can decouple mRNA and protein rhythms^20–23^—highlighting the need for complementary approaches that infer circadian relevance beyond cycling expression.

To address this gap, we developed CCorGsDB, a database of Clock Correlated Genes (CCorGs) in mouse and human CNS regions. CCorGsDB leverages Weighted Gene Co-expression Network Analysis (WGCNA) filtered by canonical clock markers to identify genes whose expression patterns are tightly coupled with circadian regulators, even in cross-sectional datasets. This resource provides a framework to explore potential circadian regulators in the CNS and to support downstream functional, pharmacological, and translational studies.

Compared to generic co-expression resources, such as the Hegemon Boolean database^24^, CCorGsDB integrates circadian biomarkers to refine gene selection, and uniquely addresses region-specific circadian regulation in the CNS. The database also integrates disease associations and CNS-targeting drugs and modulators with half-lives ≤12 h, prioritizing molecules more likely to exhibit circadian-dependent pharmacological effects within time-restricted therapeutic windows^25–27^. CCorGsDB is freely available as an interactive web platform at https://famed.ufal.br/ccorgs.

## Validation of CCorGsDB using time-series circadian datasets

**Fig. 1:**
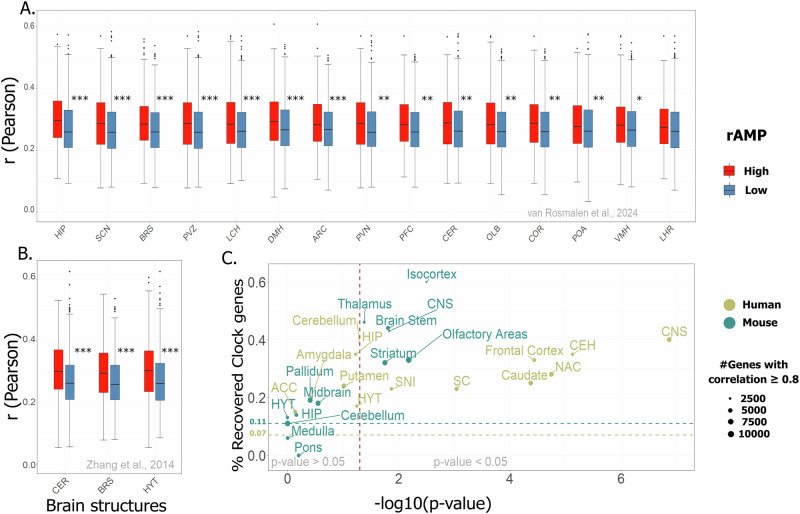
Clock correlated genes (CCorGs) are enriched with both cycling transcripts and clock genes. Correlation values were higher in the subset of genes with higher amplitudes (95th percentile) compared to those with lower amplitudes (5th percentile) across all analyzed tissues, except in the LHR (MW, *p* = 0.05259). Boxplots indicate median and 95% confidence intervals. Time course experiments were obtained from van Rosmalen et al.^17^ (**A**) and Zhang et al.^3^ (**B**). *** indicates *p* ≤ 0.001; ** *p* ≤ 0.01; * *p* < 0.05. **C** The ratio of clock genes recovered in the 90th percentile of CCorGs (with Fdr < 0.05) are higher in 13 regions compared to the mean ratio in the input samples for mice (0.11) and humans (0.07) (Fisher’s Exact Test *p* < 0.05). Vertical dashed line indicates the *p* value cutoff of 0.05. Horizontal dashed lines indicate average clock genes ratio in mouse and human input datasets for WGCNA. Supplementary Tables 1 and 2 detail the statistical parameters. relative Amplitude (rAMP); central nervous system (CNS); cerebellar hemisphere (CEH); spinal cord (SC); substantia nigra (SNI); nucleus accumbens (NAC); anterior cingulate cortex (ACC); arcuate nucleus of hypothalamus (ARC); brainstem (BRS); cerebellum (CER); whole cortex (COR); prefrontal cortex (PFC); olfactory bulb (OLB); hippocampus (HIP); dorsomedial hypothalamus (DMH); preoptic area (POA); suprachiasmatic nuclei (SCN); paraventricular nuclei of hypothalamus (PVN); lateral hypothalamus-caudal region (LCH); lateral hypothalamus-rostral region (LHR); ventromedial hypothalamus (VMH); periventricular zone (PVZ); whole hypothalamus (HYT).

We validated the database using time-series datasets from sixteen mouse CNS regions^3,17^. Genes with higher circadian amplitudes (rAMP) consistently showed stronger correlations in CCorGsDB, supporting the circadian relevance of these networks (Supplementary Table 1, Fig. 1A, B). An exception was the lateral hypothalamus-rostral region, which approached but did not reach statistical significance (*p* = 0.05259). Clock gene enrichment was also significant in multiple networks for both species (Fig. 1C, Supplementary Table 2). Notably, Per3 was the top-ranked CCorG in the human cerebellum (*r* = 0.847, *p* = 9.9e–68), while Hnrnpdl and Akt3 were the strongest correlates in mouse CNS (*r* = 0.903) and human hypothalamus (*r* = 0.919), respectively—both with known links to circadian function^28,29^.

## Cross-species identification and analyses of conserved CCorGs

To derive biological insights from the CCorGs data, we examined orthologous genes shared between mouse and human integrated CNS networks that showed strong correlations with circadian markers in both species (*r* ≥ 0.8). Protein–protein interaction (PPI) analysis using the STRING database revealed that the 251 genes that met this criterion (listed in Supplementary Data) constitute an enriched interaction network in both mouse and human (average local clustering coefficient: 0.324 and 0.381, respectively; PPI enrichment *p* < 1.0e-16), suggesting that these conserved CCorGs form non-random, functionally coherent modules rather than representing incidental co-expression artifacts.

**Fig. 2:**
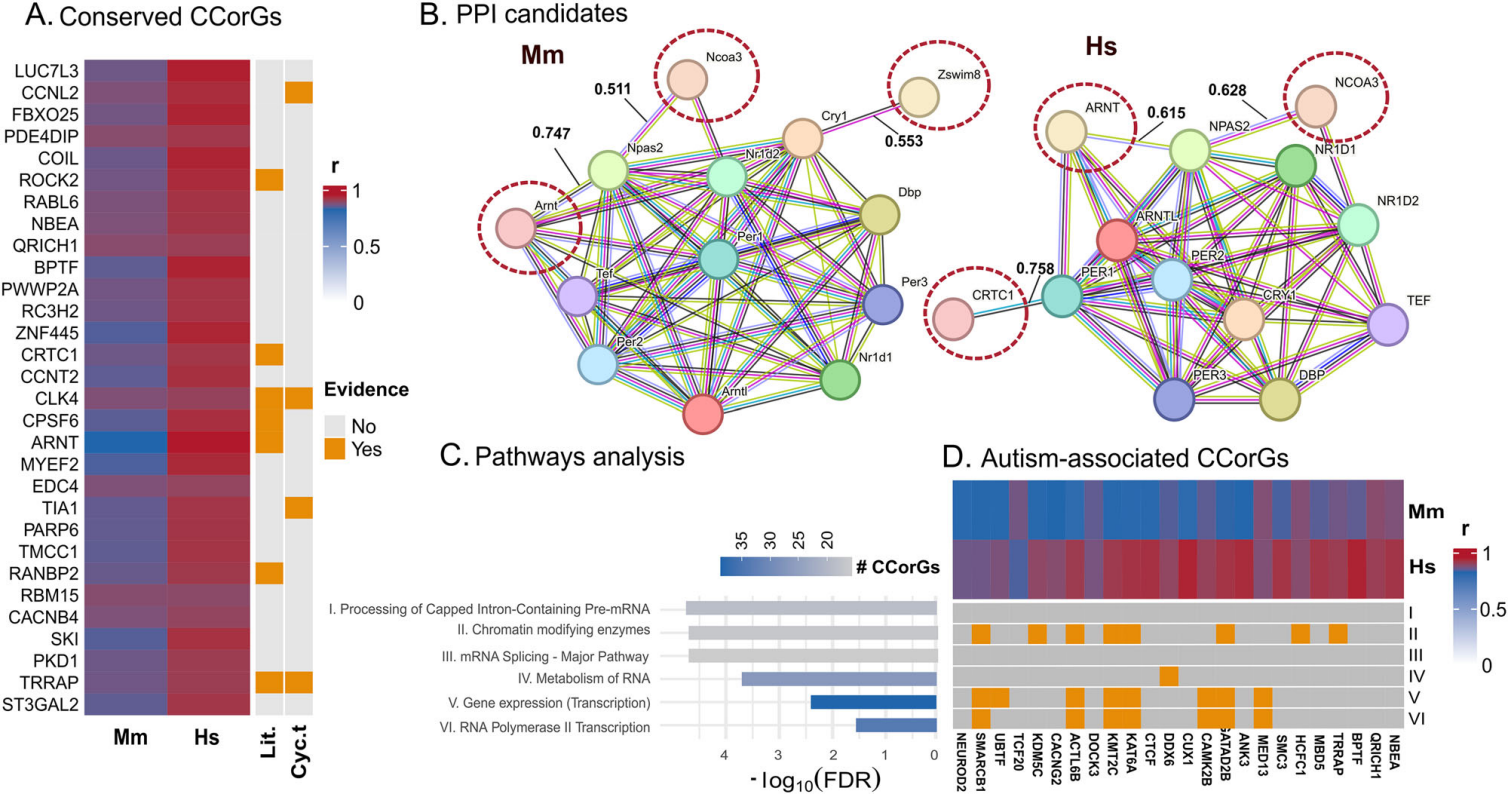
Conserved CCorGs (*r* ≥ 0.8) between mouse (Mm) and human (Hs) CNS integrated networks highlight epigenetic and post-transcriptional regulators linked to autism spectrum disorder (ASD). **A** Top 30 conserved CCorGs ranked by average Pearson correlation with canonical clock genes. Heatmap shows the highest correlation (*r*) value obtained for each CCorG. Genes displaying cycling transcript (Cyc. t.) profiles in CircaDB (JTK *q* < 0.05) among the CNS regions available in the database are indicated (Ccnl2, Clk4 and Trrap in SCN; Tia1 in hypothalamus); literature evidence (Lit.) for circadian involvement was retrieved from PubMed. **B** Candidate regulators (highlighted with dashed outlines) identified through protein–protein interaction (PPI) analysis (medium- to high-confidence STRING scores) with representative clock genes. Numbers indicate the highest combined PPI score. **C** Pathway enrichment analysis (Reactome) of the 251 conserved CCorGs showing marked overrepresentation of RNA processing and chromatin regulation pathways. **D** Conserved CCorGs associated with ASD—the most enriched Human Phenotype Ontology (HPO) term for this subset of conserved CCorGs—and their overlap with the enriched pathways shown in (**C**). Detailed results are provided in Supplementary Data.

The top 30 ranked by average Pearson correlation are shown in Fig. 2A. Among them, Rock2, Crtc1, Cpsf6, Arnt, RanBP2 and Trrap—and to a lesser extent, Clk4—have been previously implicated in circadian regulation. However, only four of the top 30 (Ccnl2, Clk4, Tia1 and Trrap) displayed rhythmic transcript profiles in CircaDB (JTK *q* < 0.05) among the CNS regions available in the database^30^, reinforcing that cycling transcripts represent only a subset of genes with circadian relevance (Fig. 2A and Supplementary Data).

Arnt, Ncoa3, Zswim8, and Crtc1 exhibit moderate-to-high combined PPI scores with canonical clock genes (Fig. 2B and Supplementary Data). Notably, Ncoa3 and Zswim8—a nuclear receptor coactivator and a ubiquitin ligase, respectively—have not been previously studied in circadian biology, positioning them as novel candidate components of clock-modulated regulatory networks. Additionally, D5ertd579e and 1700025g04rik, remain largely uncharacterized, offering further avenues for discovery.

Six genes in this conserved list (SLC8A2, PIAS1, TRRAP, MAP3K2, CNNM4, HCFC1) were reported to modulate circadian rhythms in RNAi screens in human cells (Supplementary Data)^31^. A Drosophila homolog of TRRAP has also been shown to regulate circadian rhythms through epigenetic mechanisms^32^.

Pathway enrichment analysis of the conserved CCorGs revealed significant overrepresentation of RNA processing and chromatin regulation pathways (Fig. 2C). Within these pathways, only Hnrnpm (in hypothalamus and cerebellum) and Trrap (in SCN) exhibited rhythmic transcript levels in CNS regions available in CircaDB, suggesting that many CCorGs may influence circadian processes through epigenetic or post-transcriptional mechanisms rather than via overt oscillations in mRNA abundance. These regulatory layers are well recognized as essential components of circadian timing^33,34^.

Several of these conserved genes overlap with those associated with autism spectrum disorder (ASD) (Fig. 2D), the most enriched phenotype identified in the Human Phenotype Ontology (HPO) analysis (Supplementary Data). This overlap is dominated by chromatin-associated regulators—including Trrap, Kdm5c, Kmt2c and Ctcf—alongside a single RNA metabolism factor, Ddx6, all previously implicated in circadian rhythm regulation^35–38^. This pattern suggests that disruptions in epigenetic regulatory mechanisms may contribute to the circadian alterations reported in ASD^39^.

## Disease associations and CNS-active drug targets

CCorGsDB also revealed hundreds of genes associated with other neurodegenerative and behavioral disorders, as identified via DisGeNET^40^. Some are targets of antiepileptic and dopaminergic drugs whose efficacy improves with time-specific administration^41–43^, reinforcing the value of CCorGsDB for chronotherapeutic strategies. Notably, among the conserved cross-species CCorGs, butamben—clinically used as a topical local anesthetic—was the only compound associated with this gene set through targets expressed in central neurons, mapping to Cacnb4 and Cacna1d. Both genes are linked in DisGeNET to neuropsychiatric conditions marked by circadian disruption (Supplementary Data). Although not therapeutically applied within the CNS, butamben modulates ion channels central to neuronal excitability: in experimental systems, it inhibits L-, N-, and T-type voltage-gated calcium currents and A-type potassium channels, pathways tightly coupled to circadian regulation of neuronal activity^44^. With its short half-life (~90 min), butamben may function as a mechanistically informative probe for evaluating time-of-day variation in ion-channel–dependent excitability, despite the absence of chronopharmacological studies to date.

Therefore, in preclinical settings, the database can assist in selecting genes for functional assays or testing drug timing in disease models. Clinically, it may support biomarker discovery and therapeutic optimization. Continued integration of additional data layers could further enhance its ability to predict and stratify circadian phenotypes.

## Limitations

The present study has some limitations inherent to its in silico design. Correlation with clock genes does not imply rhythmicity or functional involvement; rather, CCorGsDB identifies genes statistically coupled to canonical circadian markers, which may reflect rhythmic oscillation, direct or indirect regulation, or shared upstream control. These interpretations remain putative and require experimental and time-resolved validation. Additionally, GTEx data are subject to inter-donor variability and differences in tissue composition that were not explicitly modeled prior to WGCNA. Although module-level co-expression reduces random noise, residual confounding from donor- or batch-related factors cannot be ruled out and should be addressed in future updates of the database.

## Concluding remarks

Our approach enables the identification of candidate circadian genes across CNS regions, offering a practical alternative where time-series data are lacking. CCorGsDB extends the reach of circadian transcriptomics by capturing functionally relevant genes beyond rhythmic expression. While the findings are based on co-expression correlations and publicly available datasets, CCorGsDB provides a robust framework for hypothesis generation and future experimental validation. With its web interface, visual tools, and translational features, it serves as a resource for chronobiology, neuroscience, and molecular medicine.

## Methods

### Mouse datasets

The mouse data were obtained from the Allen Mouse Brain Atlas database (http://mouse.brain-map.org/)^45^, downloaded between 2017 and 2021. We collected the in-situ hybridization expression data (expression energy) of 15,951 genes (15,804 protein-coding) in thirteen mouse CNS tissues: brain stem, cerebellum, hippocampus, hypothalamus, isocortex, medulla, midbrain, olfactory areas, pallidum, pons, striatum, and thalamus, in both sagittal and coronal anatomical series^46^, using the ABAData R package^47^. We additionally unified the data from all tissues into a single dataset, termed the “mouse central nervous system.” For each gene in each tissue, expression data were collected from at least 20 common subregions among the total set of genes. Within this set, each gene could have more than one expression data point per sub-region, corresponding to one or more experimental replicas according to the anatomical plane and different gene sequences. The cortical subplate was not included in the analysis because it did not meet the criteria for a minimum number of subregions.

### Human datasets

The human data were collected from the Genotype-Tissue Expression (GTEx) portal (https://gtexportal.org/home/)^48^, downloaded in 2021 and 2022. We obtained the RNA-sequencing expression data of 37,464 genes (16,328 protein-coding) in twelve distinct human brain subregions of postmortem donors: anterior cingulate cortex, amygdala, caudate, cerebellar hemisphere, cerebellum, frontal cortex, hippocampus, hypothalamus, nucleus accumbens, putamen, spinal cord, and substantia nigra. We considered all samples by brain subregion, estimated at over a hundred for each of them. As was done with mouse data, we unified data from all human tissues into a single dataset, termed the “human central nervous system.”

### Construction of co-expression networks (WGCNA)

Co-expression networks were built using the “WGCNA” (Weighted Gene Correlation Network Analysis) package^49^. Twelve signed-type weighted gene co-expression networks were constructed for each species, one for each tissue, and additional networks for datasets integrating all tissues, described here as CNS networks for mice and humans. Hierarchical clustering by average linkage was implemented to detect outliers. Pearson’s correlations between each gene pair were calculated to build an adjacency matrix using the “cor” function. A soft-threshold power for each co-expression network was calculated to achieve approximate scale-free topology. Then, the topological overlap measure (TOM) and corresponding dissimilarity (1-TOM) were calculated using an adjacency matrix. 1-TOM was used as a distance for gene hierarchical clusters, and the Dynamic Tree Cut algorithm and blockwiseModules function were used to identify the modules, defined as clusters of highly interconnected genes according to their similarity of expression profiles^50^. In all networks, the minimum number of genes per module was 30, and a limit of 0.25 was used in the “cutheight” argument to determine the height at which the branches should be cut. In each module, we identified the module eigengene (ME) using the “moduleEigengenes” function, considered as a representative summary of the gene expression profile in a module and the first principal component of a given module^51^. Hub genes were determined by the highest connectivity in candidate modules, measured by Module Membership.

### Identification of circadian-associated modules

To find relevant gene modules in each co-expression network, we created module–trait relationships based on the correlation between ME and circadian clock traits. We considered traits to be the expression from ten genes recognized as the best biomarkers for circadian rhythms in 12 mouse tissues based on a machine learning algorithm: Arntl, Cry1, Dbp, Npas2, Nr1d1, Nr1d2, Per1, Per2, Per3, and Tef^27^. Eight of these are known to be core components of the molecular clock: Arntl (Bmal1), Cry1, Per1, Per2, Per3, Nr1d1 (Rev-erbα), Nr1d2 (Rev-erbβ), and Npas2^2^. Dbp and Tef are transcription factors mediating the circadian expression of many downstream genes^52^. Because each circadian gene can have one or more experimental replicas in ABA, twenty-two traits were used in total for each co-expression network construction. For each brain region, co-expression networks were constructed using between 20 and 246 samples (mouse: 20–89; human: 139–2642). The soft-threshold power (β) ranged from 6 to 12 across networks, ensuring scale-free topology (Supplementary Table 3). A correlation coefficient (*r*) ≥ 0.8 and a *p* < 0.05 were set as the criteria for significant correlation between a given ME and every circadian clock biomarker. After determining the biologically significant modules, we calculated the gene significance (GS) (the Pearson’s correlation coefficient between the gene and the circadian clock biomarkers)^27^. We then bootstrapped the correlations in 1000 simulations using the “boot” package to estimate the 95% confidence intervals.

### Validation using circadian time-series transcriptomes

We also evaluated different mouse encephalon tissues with available circadian transcriptome datasets, as a strategy to validate CCorGs as candidate genes associated with the circadian clock. From Zhang et al.^3^, we obtained brainstem, cerebellum, and hypothalamus from C57/BL6 mice. From van Rosmalen^17^, we analyzed the cortex, prefrontal cortex, olfactory bulb, hippocampus, preoptic area, suprachiasmatic nuclei, arcuate nucleus, paraventricular nuclei of hypothalamus, lateral hypothalamus-caudal region, lateral hypothalamus-rostral region, dorsomedial hypothalamus, ventromedial hypothalamus, and hypothalamic periventricular zone. In this case, only data from CBA/CaJ nocturnal mice and from equivalent regions to those present in the CCorGsDB database were used. Data normalization was performed by tissue using the DESeq2 R package^53^. The rAmp (relative amplitude) of each gene was obtained using the MetaCycle R package^54^. We used the Mann-Whitney test to evaluate the difference between Pearson correlation values in the 5th and 95th percentiles of the rAmp distribution in each region. Cohen’s *d* test was used to calculate the effect size. Additionally, we used the Fisher exact test to evaluate the possible enrichment for clock genes in a subset of CCorGs (90th percentile of GS positive correlation values—FDR < 0.05—in significant modules) based on a list of 22 genes (Arntl, Arntl2, Clock, Cry1, Cry2, Csnk1a1, Csnk1d, Csnk1e, Dbp, Fbxl3, Fbxl21, Hlf, Npas2, Nr1d1, Nr1d2, Per1, Per2, Per3, Rora, Rorb, Rorc, and Tef) which are important molecular components for the circadian clock^55,56^. We considered a 95% confidence interval and an alpha of 0.05 as a cutoff for statistical significance.

### Identification and analyses of conserved mouse–human CCorGs

To identify conserved CCorGs between mouse and human CNS networks, we first selected, for each species, all genes showing strong positive correlations with canonical circadian markers (Pearson *r* ≥ 0.8) within CNS WGCNA modules. Human gene symbols were mapped to mouse orthologs using g:Profiler (g:Orth) based on curated HGNC–MGI annotations. Only ortholog pairs meeting the *r* ≥ 0.8 threshold in both species were retained, yielding a final set of 251 conserved CCorGs (Supplementary Data). PPI analyses were performed in STRING v12.0^46^. The full conserved gene set was used as input to assess network enrichment in human and mouse, applying a medium confidence interaction score (0.400). The top 30 conserved orthologs with the highest average Pearson correlation across species were evaluated for transcript rhythmicity using CNS datasets available in CircaDB^57^. Rhythmicity was defined as JTK *q* < 0.05. Literature searches for prior circadian evidence were conducted in PubMed using each gene symbol combined with the keyword “circadian”. Pathway enrichment analysis of the 251 conserved CCorGs was performed using STRING v12.0, assessing enrichment for Gene Ontology Biological Process (GO:BP), Molecular Function (GO:MF), Reactome, Kyoto Encyclopedia of Genes and Genomes, and HPO terms, applying an FDR threshold <0.05. Additionally, we cross-referenced the conserved gene set with published RNAi-based functional screens in human cells to identify genes previously shown to modulate circadian parameters^31^.

### Disease associations and drug annotation

Neuropsychiatric disorders, cognitive, and behavioral traits associated with CCorGs were identified based on the DisGeNET database, using the disgenet2r R package^40^. Pharmacological drugs with a half-life of up to 12 h acting on the CNS were collected from the DrugBank database (https://go.drugbank.com/, v 5.1.8)^58^.

### Database implementation

The database was implemented using PHP, Javascript, HTML, JQuery, MySQL, Plotly.js, Bootstrap, and Ajax, and is available as a public domain website at https://famed.ufal.br/ccorgs. Dynamic filtering of the available datasets is provided based on different statistics and non-statistical parameters. Downloadable results for each search include images in PNG format and CSV files with reported statistics.

## Supplementary information

Supplementary information

Supplementary Data

## Data Availability

Mouse expression data were obtained from the Allen Mouse Brain Atlas (http://mouse.brain-map.org/) between 2017 and 2021. Human RNA-seq data were obtained from the GTEx portal (https://gtexportal.org/home/) in 2021–2022. Significant co-expression networks and conserved CCorGs are available at https://famed.ufal.br/ccorgs.

## Code availability

All scripts used for data processing, network construction, and correlation analyses are available upon request. The source code for the CCorGsDB web platform (frontend and backend) can also be provided by the authors for academic use. No proprietary or commercial software was used in the analyses.
