# Supplementary material for: CCorGsDB: a database for clock correlated genes in the mouse and human central nervous systems
Source: NPJ Biol Timing Sleep. 2026 Jan 9;3:3. doi: 10.1038/s44323-025-00064-y (PMC12912365; doi:10.1038/s44323-025-00064-y)
Supplement: Supplementary file 1 — Supplementary information [file 44323_2025_64_MOESM1_ESM.pdf]

Region	Corresponding area in CCorGsDB	GS mean / median in 5th percentile of rAMP (#CCorGS)	GS mean / median in 95th percentile of rAMP (#CCorGs)	Mann-Whitney p-value	Size effect (Cohen's d)
**Van Rosmalen et al., 2024**					
Arcuate nucleus	Hypothalamus	0.2723037 / 0.2667017 (998)	0.2928806 / 0.2823499 (361)	p = 0.0002941	d = 0.809557
Brainstem	Brainstem	0.267867 / 0.2592915 (998)	0.293323 / 0.2843633 (326)	$p = 3.487e^{-06}$	d = 1.037497
Cerebellum	Cerebellum	0.2749704 / 0.2620485 (979)	0.2897903 / 0.2843633 (280)	p = 0.005329	d = 0.6230717
Whole cortex	Isocortex	0.2693477 / 0.2597189 (990)	0.2903803 / 0.2866571 (250)	p = 0.00608	d = 0.7665251
Prefrontal cortex	Isocortex	0.2694226 / 0.2591665 (1087)	0.288876 / 0.2826808 (338)	p = 0.0004363	d = 0.5933874
Olfactory Bulb	Olfactory Areas	0.2693477 / 0.2597189 (990)	0.2891894 / 0.2826808 (345)	p = 0.005562	d = 0.7719158
Hippocampus	Hippocampus	0.2738964 / 0.2593759 (1015)	0.3034936 / 0.2966472 (413)	$p = 2.856e^{-09}$	d = 1.328156
Dorsomedial Hypothalamus	Hypothalamus	0.2756436 / 0.2659488 (1000)	0.2963376 / 0.2919925 (327)	p= 0.0001992	d = 0.8318283
Preoptic area	Hypothalamus	0.272095 / 0.2613014 ((1006)	0.2872428 / 0.2761689 (338)	p = 0.007576	d = 0.5971168
Suprachiasmatic Nuclei	Hypothalamus	0.2702148 / 0.2605448 (962)	0.2964105 / 0.2955416(384)	$p = 3.329^{e-07}$	d = 1.141245
Paraventricular Nuclei	Hypothalamus	0.2713585 / 0.2584326 (966)	0.2908163 / 0.2858588 (381)	p = 0.0004358	d = 0.7865114
Lateral Hypothalamus-Caudal	Hypothalamus	0.2708365 / 0.2610529 (973)	0.2928438 / 0.2847001 (374)	p = 0.0001388	d = 0.8520057
Lateral Hypothalamus-Rostral	Hypothalamus	0.2720674 / 0.2737441 (996)	0.2799556 / 0.2611548 (323)	p = 0.05259	d = 0.4334169
Ventromedial Hypothalamus	Hypothalamus	0.2732628 / 0.2648788 (980)	0.2863299 / 0.280433 (324)	p = 0.01177	d = 0.5632742
Periventricular Zone	Hypothalamus	0.269911 / 0.2585305 (981)	0.2908163 / 0.2858588 (381)	$p = 9.915e^{-05}$	d = 0.8704249
**Zhang et al., 2014**					
Brainstem	Brainstem	0.267867 / 0.2592915 (998)	0.3005153 / 0.2952658 (648)	$p = 4.58e^{-12}$	d = 1.60655
Cerebellum	Cerebellum	0.270388 / 0.2624734 (912)	0.3070353 / 0.3015766 (730)	$p < 2.2e^{-16}$	d = 1.879093
Hypothalamus	Hypothalamus	0.2742063 / 0.2621772 (887)	0.3043939 / 0.3038627 (649)	$p = 3.239e^{-11}$	d= 1.483688

Supplementary Table 1. Mann-Whitney test was conducted to evaluate the difference between Gene Significance (GS) values in the 5th and 95th percentiles of the rAMP distribution in each region analyzed from Van Rosmalen, et al., 2024 [62] and Zhang et al., 2014. Cohen's d test was used to evaluate the size effect.

Transcriptome	# Input genes	# Clock genes	% Clock genes	# CCorGs (90th percentile)	# Recovered Clock genes	% Recovered clock genes	
**Mouse**							
Isocortex	19933	22	0.11	1124	6	0.53	p = 0.003185*
Olfactory Areas	19933	22	0.11	5162	15	0.29	p = 0.006571*
Central Nervous System	19933	22	0.11	1588	6	0.37	p = 0.0149*
Brain Stem	19933	22	0.11	2058	7	0.34	p = 0.01557*
Striatum	19933	22	0.11	4006	11	0.27	p = 0.01765*
Thalamus	19924	22	0.11	2054	8	0.38	p =0.04183*
Midbrain	19933	22	0.11	5452	9	0.16	p = 0.2816
Pallidum	19923	22	0.11	3047	5	0.16	p = 0.3937
Pons	19932	22	0.11	1219	0	0	p = 0.6352
Hippocampus	19932	22	0.11	1609	2	0.12	p = 0.6994
Hypothalamus	19885	22	0.11	980	1	0.10	p = 1
Cerebellum	19924	22	0.11	4217	4	0.11	p = 1
Medulla	19932	22	0.11	1662	1	0.06	p = 1
**Human**							
Central Nervous System	53921	22	0.04	5015	15	0.29	p = 1.367e-07*
Cerebellar Hemisphere	50943	22	0.04	3084	10	0.32	p = 7.479e-06*
Nucleus Accumbens	51506	22	0.04	4944	12	0.24	p = 1.786e-05*
Frontal Cortex	51004	22	0.04	3038	9	0.29	p = 3.671e-05*
Caudate	51311	22	0.04	5415	12	0.22	p = 4.221e-05*
Spinal Cord	50064	22	0.04	3822	8	0.20	p = 0.0009127*
Substantia Nigra	49596	22	0.04	1858	4	0.21	p = 0.01351*
Cerebellum	17526	22	0.12	1015	4	0.39	p = 0.05149
Hypothalamus	51222	22	0.04	1882	3	0.16	p = 0.05739
Hippocampus	17549	22	0.12	1993	6	0.30	p = 0.05978
Putamen	17521	22	0.12	3702	9	0.24	p = 0.09748
Amygdala	17528	22	0.12	2478	5	0.20	p = 0.3725
Anterior Cingulate Cortex	17958	22	0.12	1933	3	0.15	p = 0.7301

Supplementary Table 2. Fisher's exact test was used to compare clock gene ratios in the 90th percentile of positive correlations (FDR < 0.05) from the CCorGsDB by tissue in the mouse and human CNS with the frequency of clock genes found in the mouse and human transcriptomes used as input for WGCNA.

Tissues	Number of Samples	Number of Input genes	*Soft-Threshold Power* (β)	r_{min}	r_{max}	Mean	SD
**Mouse**							
Central Nervous System	89	19933	8	0.0020	0.9036	0.55	0.17
Isocortex	62	19933	6	0.0072	0.9344	0.58	0.16
Hippocampus	33	19932	7	0.0026	0.9694	0.66	0.17
Medulla	30	19931	8	0.0009	0.9867	0.68	0.15
Midbrain	22	19933	8	0.0003	0.9917	0.69	0.15
Brain Stem	22	19933	7	0.0001	0.9820	0.65	0.19
Pallidum	21	19923	8	0.0074	0.9961	0.72	0.16
Thalamus	21	19924	8	0.0001	0.9617	0.56	0.18
Olfactory Areas	20	19933	10	0.0005	0.9914	0.71	0.16
Cerebellum	20	19924	9	0.0006	0.9928	0.74	0.17
Pons	20	19932	8	0.0060	0.9770	0.71	0.15
Striatum	20	19933	8	0.0008	0.9916	0.69	0.17
Hypothalamus	20	19885	6	0.0034	0.9931	0.70	0.16
**Human**							
Central Nervous System	2642	53921	6	0.0006	0.9595	0.61	0.18
Anterior Cingulate Cortex	176	17958	8	0.0007	0.9552	0.67	0.17
Amygdala	152	17528	12	0.0032	0.9179	0.64	0.13
Caudate	246	51311	10	0.0009	0.9659	0.58	0.19
Cerebellar Hemisphere	215	50943	8	0.0003	0.9331	0.56	0.17
Cerebellum	241	17526	9	0.0513	0.8476	0.58	0.14
Frontal Cortex	209	51004	8	0.0014	0.9409	0.58	0.19
Hippocampus	197	17549	8	0.0011	0.9363	0.62	0.17
Hypothalamus	201	51222	10	0.0060	0.9188	0.59	0.17
Nucleus Accumbens	246	51506	8	0.0003	0.9660	0.58	0.20
Putamen	205	17521	10	0.0059	0.9722	0.69	0.15
Spinal Cord	159	50064	10	0.0005	0.9253	0.55	0.22
Substantia Nigra	139	49596	8	0.0076	0.9273	0.60	0.19

Supplementary Table 3. Mouse and human tissues, input parameters used for Weighted Gene Co-expression Network Analysis (WGCNA) and overall positive correlations for CCorGs sets within networks. Mean, SD (standard deviation), rmin (minimum GS Pearson correlation), rmax (maximum GS Pearson correlation).

Supplementary Data. Excel file containing the 251 conserved CCorGs shared between mouse and human CNS integrated networks (Pearson r ≥ 0.8 in both species), identified through orthology mapping (g:Profiler g:Orth). For each gene, the file includes protein annotation; STRING-derived pathway enrichment results (GO Biological Process, GO Molecular Function, Reactome, KEGG); and Human Phenotype Ontology (HPO) terms. Additional columns include protein–protein interaction metrics (highest combined PPI score and interacting clock-gene partner), as well as disease and drug associations derived from DisGeNET and DrugBank, respectively. The RNAi column indicates genes reported to affect circadian parameters in cultured-cell screening assays, with the corresponding phenotypes.

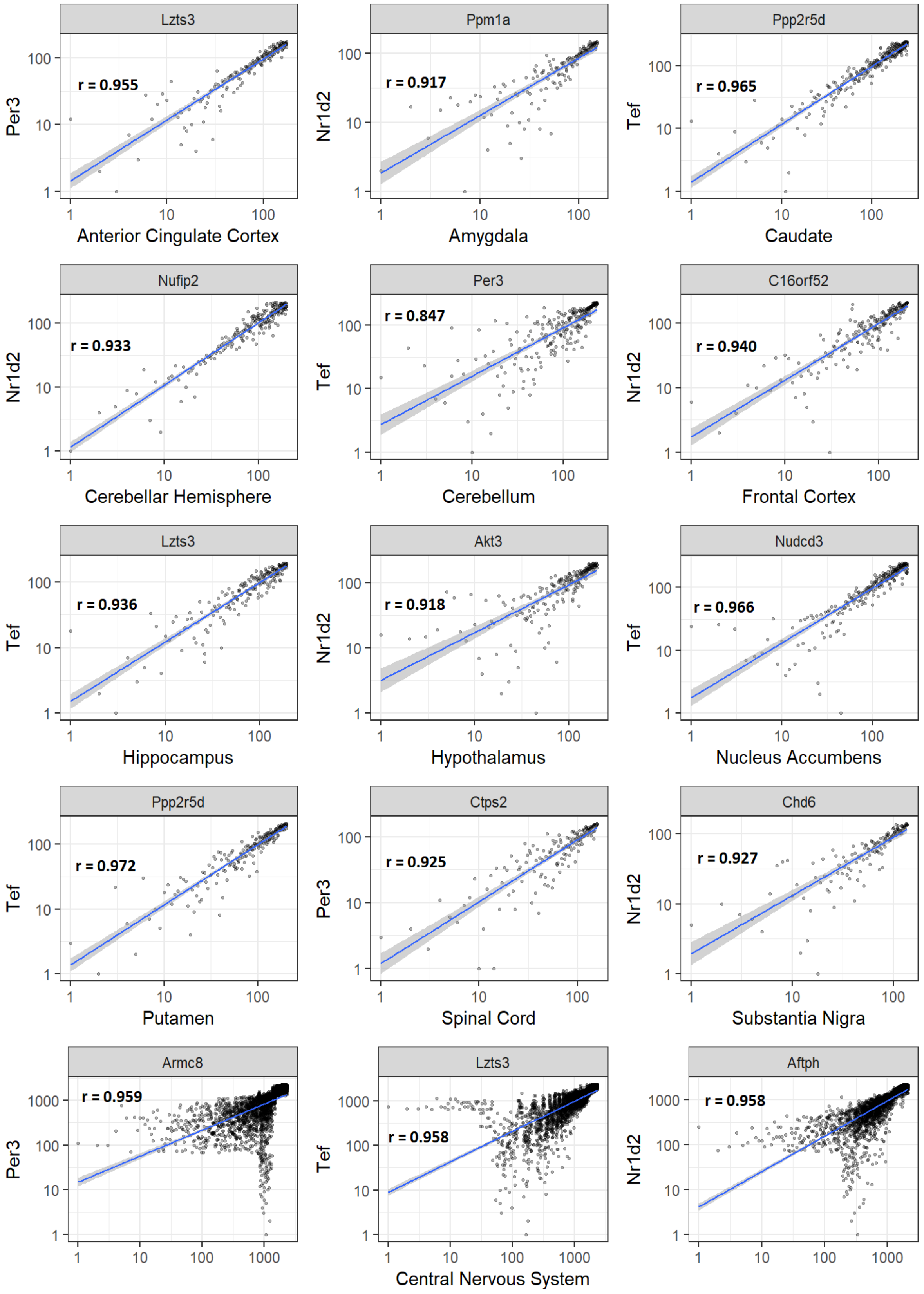

Supplementary Figure 1. Top Clock Correlated Genes (CCorGs) by each tissue and the top 3 CCorGs in the integrated Central Nervous System networks, with their respective correlated clock genes in the human data. r = Pearson correlation value.

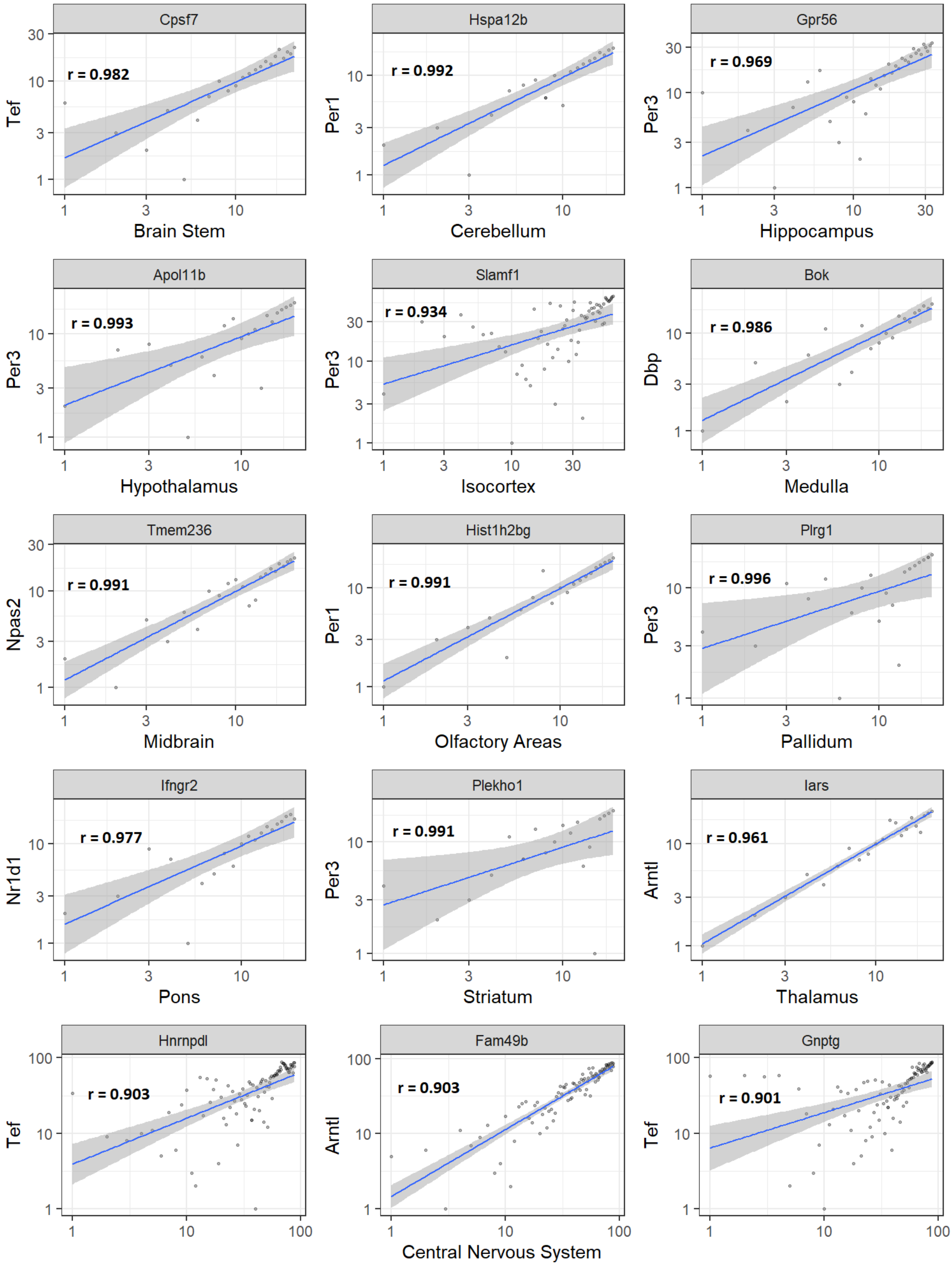

Supplementary Figure 2. Top Clock Correlated Genes (CCorGs) by each tissue and the top 3 CCorGs in the integrated Central Nervous System networks, with their respective correlated clock genes in the mouse data. r = Pearson correlation value.
